# Organogels for the Preservation of Cultural Heritage

**DOI:** 10.3390/gels11090715

**Published:** 2025-09-05

**Authors:** Damiano Bandelli, Céline Adamo, Giovanna Poggi, David Chelazzi, Piero Baglioni

**Affiliations:** Department of Chemistry, University of Florence and CSGI, Via della Lastruccia 3, 50019 Sesto Fiorentino, Italy; damiano.bandelli@unifit.it (D.B.); celine.adamo@unifi.it (C.A.); giovanna.poggi@unifi.it (G.P.)

**Keywords:** organogels, cleaning paintings, cultural heritage conservation, sustainable gels, green gels

## Abstract

The degradation of works of art, enhanced by climate change, needs to be counteracted to have Cultural Heritage express its full socioeconomic potential. Cleaning artifacts requires the confinement of fluids in retentive gel matrices to achieve safe, time-effective removal of soil, aged coatings, or vandalism from artistic/historical surfaces. This review discusses past and current research in organogels, which are largely unexplored systems to confine average or low polarity solvents. Particular focus is on bio-derived, “green”, and sustainable materials, polymers, and solvents. Perspectives in this field strongly link with current recommendations for sustainable design in materials science and multiple industrial sectors.

## 1. Introduction

Over the last decade, our society has been facing growing socioeconomic challenges, condensed in global energy crisis and the effects of climate change. These issues pose serious threats to our society’s resilience, urgently calling for sustainable solutions, where materials science can give valuable contributions [1,2,3]. In this context, the preservation of Cultural Heritage represents a crucial framework where important assets are endangered, and new advanced materials are required. Degradation processes constantly affect works of art, typically starting at the works’ surface, caused by environmental factors, pollutants, vandalism, and even wrong interventions [4,5,6]. Degradation directly affects the physical integrity of the artworks, their accessibility, and thus their socioeconomic value and their transfer to future generations. In response, research has produced several classes of advanced functional materials, targeting the removal of soil and other unwanted layers (cleaning) [7], the mechanical strengthening of artistic substrates (consolidation), or the application of surface coatings against contaminants and degradation agents (protection) [8,9,10], as well as regulators and absorbers of pollutants, or sensors for monitoring atmospheres in enclosures (prevention) [11,12,13,14,15]. These solutions have twofold value since they preserve Cultural Heritage but can also, in some cases, be transferred to other sectors where smart, responsive, and energy-effective materials are constantly needed, spanning from healthcare to the food, cosmetic, and building industry [16,17,18,19].

In particular, the cleaning of artworks is a recurring and delicate task where polymer science, soft matter, and colloids have been providing sophisticated systems with optimal performances, such as nanostructured fluids (micellar solutions and microemulsions) and polymeric gels [7,20]. Nanostructured fluids have improved over classic solvent blends of the traditional restoration practice by dispersing organic solvents as dynamic nanosized micelles in continuous aqueous phases, leveraging on physico-chemical processes different than classic solvency to remove soil or aged coatings, such as the selective dewetting or swelling/detachment of unwanted layers from the original artistic surfaces [7,20,21]. However, in most cases, it is necessary to confine cleaning fluids in retentive matrices to regulate their action at the artworks’ surface, boosting their selectivity and avoiding risks to water- or solvent-sensitive surfaces. Therefore, research has devised several families of polymeric hydrogels, with chain networks held either by covalent (chemical gels) or secondary bonds (physical gels) able to confine aqueous solutions of surfactants, chelating agents, acids/bases, and enzymes or oil-in-water fluids [20,22,23].

Gels overcome the limitations of traditional polymeric solvent thickeners, providing enhanced control on the release of uploaded fluids and optimal mechanical properties that allow the feasible handling and removal of the cleaning systems after their application. A recent representative example regards the new class of “twin-chain” polymer hydrogel networks of polyvinyl alcohol, where the possibility of controlling the micro- and nano-porosity and tortuosity of the gel networks regulates the action of fluids at the gel-target interface. This feature has allowed the safe and time-effective restoration of masterpieces such as works by Pablo Picasso, Jackson Pollock, Rory Lichtenstein, and others, which would have been too risky or time-consuming with conventional solution or thickener chemistry [24].

However, the restoration of works that cannot tolerate even the minimal contact with water, such as, for instance, some acrylics or modern oils [25,26,27,28], and the exploration of novel classes of “green” solvents with intriguing physico-chemical properties [7,29,30,31] are urgently calling for the development of new organogels, complementary to the most sophisticated hydrogels currently available to restorers and curators. Research is still in its infancy, with numerous possibilities still unexplored in the synthesis and physico-chemical characterization of organogels’ structures and dynamics. In addition, compliance with environmental and health policies calling for sustainable and “green” materials, like the Green Deal, is urging for the development of Safe and Sustainable by Design approaches (SSbD) to devise energy- and time-effective materials with low ecotoxicological impact and feasible upscale [32,33,34,35]. To address this challenge, fundamental inspiration comes from nature itself, pointing to bio-based and bio-derived systems to replace petroleum-based chemistry [36,37].

In such a context, this review provides an up-to-date overview on the organogel systems devised for the cleaning of works of art, starting from the traditional solutions up to cover the first organogels tailored to remove aged coatings, and then illustrating the latest research on bio-derived polymeric gels, adding some perspectives on research in this challenging and exciting scientific and technological field. In addition to remarking the importance of novel tools for the conservation of works of art, we also hope that this review can serve as an inspiration for developing advanced materials and transferring them to multiple scientific and technological sectors even beyond Cultural Heritage preservation, fostering sustainable socioeconomic regrowth.

## 2. Traditional Polymeric “Solvent Gels” and First Organogel Formulations

The traditional practice in cleaning works of art is still mostly based on solution chemistry, where solvent cleaning follows the principle of “like dissolves like”, meaning a solvent with similar polarity to the unwanted material is used to dissolve it, while trying to minimize interactions with the underlying artistic surface. However, the substrate is not completely inactive, so some level of interaction with the solvent is often expected. The selection of a suitable solvent is typically based on its solubility parameters [38,39] and by empirical tests designed to assess the polarity of the substances to be removed, such as the Feller test and contact angle [39,40]. In particular, solubility parameters are essential to select the solvent required for the removal of organic varnishes. Briefly, the total solubility parameter (*δ*_tot_) of a compound corresponds to its cohesive energy density as defined by Hildebrand [41]. Hansen divided such quantity into three contributions, namely, dispersive (*δ*_d_), polar (*δ*_p_), and hydrogen bonding (*δ*_h_), which, for vaporizable compounds such as solvents, can be expressed as follows [42]:

(1) $$\delta_{tot}=\sqrt{\frac{{∆H}_{vap}-RT}{V}}=\sqrt{\delta_{d}^{2}+\delta_{p}^{2}+\delta_{h}^{2}}$$

where Δ*H_vap_* is the molar enthalpy of vaporization, *R* is the gas constant, *T* is the absolute temperature, and *V* is the molar volume. The quantity Δ*H_vap_−RT* corresponds to the compound’s cohesion energy.

Such approach enables the calculation of solubility parameters for solvents and small molecules, while polymers require further evaluation of their solubility in solvents with known *δ* values, defining a solubility zone by indicating which values of *δ_d_*, *δ*_p_, and *δ*_h_, and, in turn, which solvents, are capable of dissolving or swelling the target polymer. This approach enables the calculation of the target polymer’s solubility according to the following [39]:

(2) D=4δd,S−δd,P2+δp,s−δp,P2+δh,S−δh,P212

where *D* is the difference in the *δ*_d_, *δ*_p_ and *δ*_h_ values between the polymer and solvent, and *S* and *P* are related to the solvent and polymer species, respectively.

Moreover, the Teas fractional representation (*f*) allows a two-dimensional representation of Hansen solubility parameters according to

(3) $$f_{x}=\frac{\delta_{x}}{\sum_{i=0}^{3}\delta_{i}}$$

where *x* and *i* are one of the three *δ* components, namely, dispersive, polar, or hydrogen bonding [39]. As a result, a triangular plot of dispersive, polar, and hydrogen bonding interactions can directly match the solubility of different compounds.

While this approach has practical use and simplicity, it can be inaccurate to describe solvent interactions with molecules and polymers since it ignores the equilibrium between entropy-driven swelling by osmotic pressure forces and elastic pressure in polymers, included for instance in the Flory–Rehner model, which can be determinant to model the swelling of paint layers by solvent blends [43,44,45]. In addition, Baij et al. [46] recently provided a “diffusion-swelling” model to describe solvent penetration through oil paint layers considering the solvent diffusion coefficient as an exponential function of the polymer volume fraction, rather than adopting a limited Fickian diffusion model or more comprehensive (but not easy to access experimentally) free-volume models. Accordingly, solvents that swell oil layers (e.g., acetone) show faster diffusion than weakly swelling solvents (water and cyclohexane). Overall, the final recommendation was to develop confining systems for aqueous or aliphatic hydrocarbons cleaning fluids, or for microemulsions of these compounds.

Such recommendation supports the continuous research carried out in the conservation science community on gels to control the action of fluid cleaning systems. Conservators have increasingly recognized gels as a valuable improvement on traditional solvent-based methods for the removal of varnishes and overpaints. Their capability to confine and release a target solvent in a controlled manner enables greater control of layers removal and reduces risks related to solvent cleaning.

In the 1980s, Wolbers proposed the so-called “solvent gels”, which are viscous polymer dispersions, rather than true gels, to thicken solvent blends and reduce the risks associated with solvent cleaning, such as the leaching of polar materials from oil paints [47,48]. These formulations are typically prepared using polyacrylic acid (PAA) thickened with a weakly basic non-ionic cocoamine surfactant (Ethomeen^®^, Nouryon, Amsterdam, Netherlands). Ethomeen^®^ surfactants are available with different hydrophilic–lipophilic balance (HLB) values, enabling the thickening of a wide range of solvents, from low to high polarity, thus making solvent gels highly versatile and accessible tools used on numerous different artworks from metal to stone and canvas/easel paintings, etc. [49]. Solubility testing and solubility parameter-based predictions are applied to select single solvents or blends that can swell or dissolve the undesired layers. Ideally only the unwanted layers are affected; however, selectivity is often limited, so polymers are used to thicken the solvents and to gain spatial and temporal control during cleaning. Nevertheless, removing these polymer dispersions from treated surfaces typically requires the use of clearing solvent blends after application, which can be invasive to the paint layers and induce changes in the surface [50], and FTIR, SEM-EDS, and GC-MS were employed to evaluate how effectively solvent gels limit leaching from the original binder. Moreover, the limited retentiveness of solvent gels can restrict their application on substrates that are particularly sensitive to solvents. Further concerns also regard the toxicity of the cocoamine surfactants [7] and their possible degradation to reactive amine N-oxide that can alter terpenoid resins and oil-based binders commonly found in paintings [51].

To address these limitations, researchers have been developing over the last decade highly retentive gel matrices with enhanced mechanical properties, enabling residue-free removal without the need for rinsing solvents. Such retentive networks have shown successful confinement of various liquid systems, resulting in the preparation of both hydrogels and organogels. Efficient confinement within gel matrices also significantly reduces solvent volatility and toxicity to operators. Research has initially focused on hydrogels, which can confine and gradually release a wide range of aqueous cleaning solutions and are still extensively studied [52,53]. In parallel, over the past decade, organogels have attracted increasing interest due to their ability to confine organic solvents with lower polarity than water, representing a complementary tool to hydrogels for the cleaning of surfaces that cannot tolerate even the minimal contact with water. Some examples include paper artworks with water-soluble inks or dyes and certain formulations of acrylic or oil paintings [54]. We report here on the first organogels developed for applications in Cultural Heritage preservation, presented in chronological order and categorized by material type. Organogel networks can be formed either by polymeric and low-molecular weight gelators via a polymerization reaction or self-assembly, with the target of minimizing residues after cleaning artistic surfaces [55].

A first formulation involved isothermally (room temperature) rheoreversible organogels that can rapidly switch from a gel to a solution state. The rationale was to apply the gel directly onto painted surfaces, leave it in contact for the required minimum time, and then easily remove it by inducing a chemical perturbation that converts the gel into a low-viscosity fluid. This class of gels is made from polyallylamine-derived (PAA) polymeric gelators, whose organic solvents solutions become gels upon the addition of CO_2_ that converts two amino groups into an ammonium/carbamate pair. The gel–sol transition of this “smart” organogel is then triggered by the addition of a weak acid, which displaces CO_2_ and converts the carbamate groups back into cationic ammonium groups [55,56]. However, since PAA is unstable in its neutral form, an alternative polymer, polyethyleneimine (PEI), was investigated with good results [57]. These systems removed dammar from an aged 19th-century painting; from an Italian 15th-century oil-on-wood panel, removed an acrylic resin from Renaissance murals in the Santa Maria della Scala Sacristy in Siena (Italy); and cleaned a 14th-century work at the National Gallery of Siena. FTIR analyses demonstrated good cleaning efficacy. Before extensive use on easel paintings or secco murals, it is important to determine application times and procedures, also using solubility parameters, to guarantee selectivity toward the patinas to be removed, and to assess the amounts of polymer that could diffuse into and remain within the paint layers.

While this approach has potential applicative interest, successive research has focused on the synthesis of organogels with high viscoelasticity to apply and remove in one step without leaving polymer residues on the treated surfaces. To this purpose, a class of chemically crosslinked polymeric organogels based on poly(methyl methacrylate) (PMMA) or poly(ethyl methacrylate) (PEMA) was proposed [49,58].

PMMA-based organogels are typically obtained via free radical copolymerization of methyl methacrylate (MMA) with diacrylate monomers in various organic solvents, such as esters (e.g., ethyl acetate and butyl acetate) or ketones (e.g., methyl ethyl ketone, MEK). The selected solvents employed for the synthesis should span over a range of average polarity, which makes them suitable for dissolving or swelling a variety of natural and synthetic resins frequently encountered in conservation treatments [54]. MMA was solubilized in different pure organic solvents, namely, methyl ethyl ketone (MEK), cyclohexanone (cyclo), ethyl acetate (EA), and butyl acetate (BA). These were chosen for their intermediate polarity (e.g., by Teas solubility parameters), making them versatile for dissolving or swelling a wide range of natural and synthetic resins frequently removed by conservators.

For instance, a PMMA–MEK-based organogel was developed by tuning the crosslinker content and monomer–solvent ratio to enhance retentiveness and mechanical properties, ensuring a controlled solvent release suitable for cleaning solvent-sensitive substrates such as inked paper [59]. This opened a series of formulations to address a common challenge for paper conservators, i.e., the removal of aged pressure-sensitive tapes (PSTs) from contemporary drawings.

**Figure 1:**
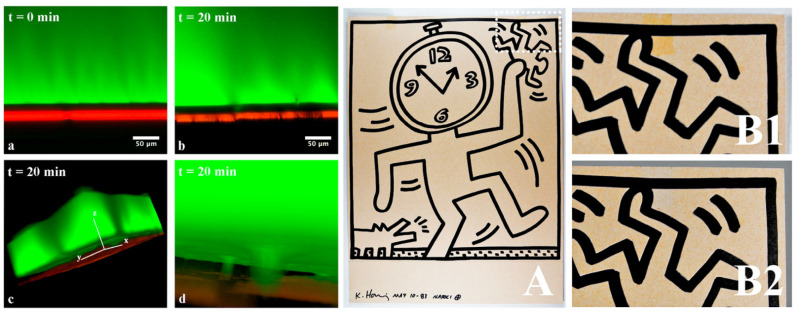
(**Left**). Laser scanning confocal microscopy measurements showing the interaction between a PEMA organogel loaded with diethyl carbonate, marked green, and the acrylic adhesive of a polypropylene-pressure sensitive tape, marked red. The black layer corresponds to the polypropylene backing of the tape. After application (time zero), the two fluorescent probes are well separated (**a**), while after 20 min, an evident penetration of the green dye through the backing is visible (**b**–**d**). (**Right**). Untitled, K. Haring, 1983—recto of the artwork (**A**). The dashed rectangle shows the area a stain of an aged pressure sensitive tape on the verso-side of the drawing. Details of the stain before (**B1**,**B2**) after cleaning. Reproduced with permission from [58]. © 2018 Elsevier Masson SAS. All rights reserved.

To address this issue, PMMA- and PEMA-based networks, prepared via radical polymerization, were swollen in alkyl carbonates, an environmentally friendly class of solvents, such as diethyl carbonate (DEC). Thanks to the entrapment of the solvent within the gel matrix, the organogels can be effectively applied to the surface of the PST, gradually penetrating its plastic layer and swelling the adhesive. These organogels selectively removed wax residues from a 19th-century inked document without altering original materials and removed natural and synthetic varnishes from easel paintings without detected damage or residues. In addition, these systems gave good results for the removal of aged pressure sensitive tapes from paper artworks, allowing for the complete removal of aged polypropylene backing tapes from a drawing by Keith Haring [58] (Figure 1) and the removal of the PTS adhesive layer from different drawings by Federico Fellini of the film library of Rimini [60]. ATR-FTIR measurements verified the cleaning performance of PMMA organogels, confirming the complete removal of varnish layers and the absence of gel residues on the treated surfaces.

**Figure 2:**
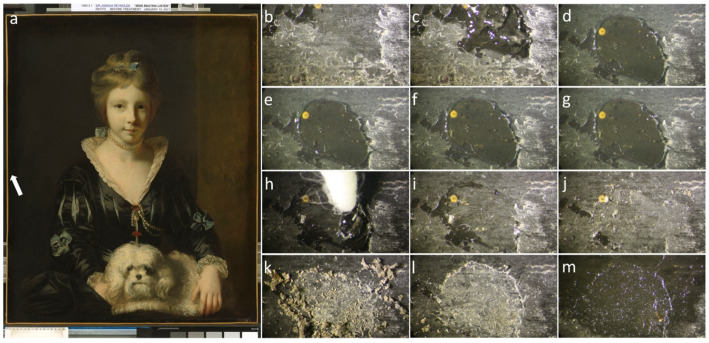
(**Left**). Sir Joshua Reynolds, Miss Beatrix Lister, 1765, National Gallery of Art, Washington (**a**), the arrow indicates the testing area, DC. (**Right**). Removal of a shellac layer with a 6 wt % 40PVAc/0.3 wt % BDBA in 95:5 ethanol/water gel. The images have been extracted from a movie of the cleaning process and shows the spot before the cleaning (**b**), the gel during the application at different contact times (**c**–**g**), the cleaning process using cotton swab (**h**–**j**), the removal of dried shellac (**k**,**l**), and the surface after the celling, coated with mineral spirit to mimic how the surface is expected to look after application of fresh varnish (**m**). Reprinted with permission from [61]. Copyright © 2017, American Chemical Society.

While these rigid organogels are suitable for application on flat surfaces, highly viscous polymer dispersions (HVPDs) are a class of materials to target the cleaning of delicate and textured surfaces. These HVPDs are composed of 40% of hydrolyzed poly(vinyl acetate) (40PVAc) and benzene-1,4-diboronic acid (BDBA) as the crosslinker, dissolved in organic solvents such as DMSO, DMF, THF, methanol, and 2-ethoxyethanol [61,62,63,64]. The obtained dispersions can be applied onto surfaces and then be easily removed after treatment simply by peeling (Figure 2). However, it must be observed that these systems do contain small amounts of water, produced by the formation of crosslinks. For this reason, Duncan et al. decided to employ two additional crosslinkers of 40PVAc: benzene-1,3-diboronic acid (1,3-BDBA), chosen to introduce nonlinear crosslinks, and biphenyl-4,4′-diboronic acid (bPDBA), used to form longer and more flexible crosslinks. These HVPDs exhibit good stability that makes them promising candidates for applications including the controlled softening and removal of varnish layers from painted artworks [65]. Indeed, they have been tested on several types of artworks. On a 16th-century gilded reliquary with painted gold leaf, they removed oxidized varnish, leaving no detectable residue. On an 18th-century canvas painting (1765 “Miss Beatrix Lister” by Sir Joshua Reynolds), these organogels swelled aged shellac varnish, enabling its safe mechanical removal without paint damage, while on other 16th- and 18th-century oil paintings they allowed softening and the removal of solvent-resistant coatings while preserving the paint layer. On marble with artificial brochantite and antlerite, they allowed reducing copper corrosion without harmful residues.

**Figure 3:**
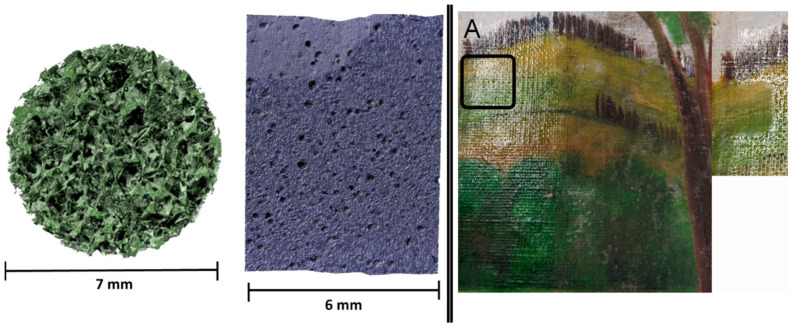
(**Left**). X-ray micro-tomography images of PDMS sponges obtained using two different templating agents, which leads to different porosity and structure. (**Right**). Removal of a 25-year naturally aged polymeric ketone resin varnish from an easel painting mock-up using a PDMS system loaded with 10 wt% of ethyl acetate; (**A**) shows the testing area. Reproduced from [68]. Copyright © 2023, The Authors.

Recently, several research studies have focused on polymeric materials with porous structures, such as porous organic polymers (POPs), due to their large surface areas and well-defined, tunable pore size distributions. These materials are promising candidates for various technological applications, including energy storage, drug delivery, and tissue engineering [66]. One example of a POP is polydimethylsiloxane (PDMS), an organogel sponge known for its elasticity, controllable porosity, and low toxicity [67]. Porpora et al. recently proposed PDMS organogels in the field of cultural heritage conservation [68]. In their work, the authors focused on the porosity of PDMS sponges, which can be tuned by varying the granularity of a templating agent (Figure 3). They also tested the combability of the PDMS networks with a wide range of mid-to-low polarity organic solvents. Two distinct templates, sugar cubes and powdered sugar, yielded sponges with significantly different pore structures: the former exhibited high porosity (77%) and a large mean pore size (300 µm), while the latter showed lower porosity (10%) and smaller pores (75 µm). The characterization of the synthesized sponges confirmed the absence of sugar residues after preparation. PDMS sponges demonstrated controlled solvent absorption and release, with higher porosity leading to greater absorption capacity and faster uptake rates. Absorption depends on solvent polarity: low-polarity solvents (e.g., cyclohexane) were absorbed more efficiently than highly polar ones (e.g., water and DMSO) in line with the hydrophobic nature of PDMS. The swelling capacity of PDMS-based systems is also strongly influenced by porosity. Non-porous PDMS slabs swell only through chain relaxation and solvent diffusion, whereas porous sponges also benefit from capillary uptake within their pores. Low-polarity solvents swell faster and reach higher maxima due to strong hydrophobic interactions with the PDMS network, while high-dielectric solvents show reduced and slower swelling. Overall, porous PDMS combines matrix swelling with capillary-driven uptake, enabling higher loading and faster kinetics than non-porous PDMS.

Application tests on fresco and canvas mock-ups successfully demonstrated the ability of powdered sugar-derived PDMS sponges loaded with ethyl acetate to selectively remove aged polymeric coatings, restoring the original surface morphology without leaving any detectable PDMS residues. In particular, tests included the removal of a 20-year-old poly(EMA/MA) 70:30 surface layer from a fresco mock-up and of a 25-year naturally aged polymeric ketone resin varnish from an easel painting mock-up. FTIR of the cleaned surface confirmed that the aged polymer layer was no longer detectable after a 12-min treatment with the PDMS, evidenced by the disappearance of the 1735 cm^−1^ carbonyl stretching band, a marker of poly(EMA/MA) 70:30. These findings strongly support the potential of PDMS organogel sponges as innovative, controlled, and gentle cleaning tools for a wide range of painted surfaces in art conservation.

Table 1 summarizes the mechanical properties, loadable solvents, cleaning efficiency, and tested case studies for the polymer dispersions and traditional organogels discussed in this session. Overall, all the organogels and HVPDs discussed above represent significant improvements over traditional solvent blends and solvent gels [69]. However, the growing requirements for fully “green” formulations in scientific and technological applications [70,71,72] have progressively involved also conservation science, which can indeed act as a driver for the development of new sustainable systems, potentially useful also to other fields such as the cosmetic, food, detergency, and healthcare industry. Therefore, bio-derived and bio-based materials are gaining growing interest in the formulation of gelled systems for art cleaning, complementing or completely replacing petroleum-based chemistry or materials with inherent or potential toxicity [73,74,75]. Accordingly, the following section will report on the latest research in “green” and sustainable organogel formulations for the conservation of Cultural Heritage.

## 3. New Sustainable Organogels for Cultural Heritage Preservation

Nowadays, the research, design, and development of new sustainable materials represent a key point in all chemistry and material-science-related fields since it enables the improvement of materials’ properties and activities [76]. A vast landscape of research fields comprising Cultural Heritage can strongly benefit from the design of new materials and their in-depth study. In this context, bio-based or bio-derived sustainable organogels represent a class of materials featuring optimum compatibility with organic solvents that finds ideal applications for the cleaning of water-sensitive surfaces and/or for the removal of hydrophobic protectives, with potential transfer to other technological or industrial sectors [20,77,78,94,95,96]. Possibilities are numerous as the type of bio-derived materials is changed and matched with different solvents. For instance, considering physical organogels from vegetable and mineral oil [78], the minimal gelling concentration (MGC) is higher in vegetable than in mineral oil. This value corresponds to the minimum amount of crystals needed to form a self-supporting network and depends on the gelator’s solubility, which is influenced by the time–temperature history. As gelator–solvent interactions increase, gelator solubility also increases, so higher gelator content is required to build a self-supporting crystalline network.

Indeed, in addition to a wide range of properties that can be tuned to improve the final material’s performances, parameters such as renewability, energetic efficiency, and toxicity of the preparation step are becoming pivotal for the assessment of new “green” materials in different research/industrial fields such as CH, making the development of environmentally friendly products of outmost importance for material’s design. For instance, further toxicological studies are necessary to confirm the safety of cinnamic acid at certain concentrations in physical gels rice bran oil and cinnamic acid [77]. These are fundamental requirements to improve on traditional thickeners such as the “solvent-gels” or siloxane and dimethicone cross-polymers [97] (e.g., Velvesil Plus™, Momentive Inc., New York, NY, USA), or the HVPDs and organogel formulations discussed in the previous section. Given the vast list of properties and features that can be varied during materials’ development such as gel’s type (i.e., physical or chemical) and reactants/constituents (monomers, polymers, and solvent employed), possibly an infinite list of materials can be obtained. It follows that there is a strict necessity to select features of interest to guide fast and effective material’s development. For instance, Passeretti et al. reported an overview related to bio-derived gels for applications in metallic CH, hinting toward to the central role of the solvent encapsulated in the gel matrix [98].

**Figure 4:**
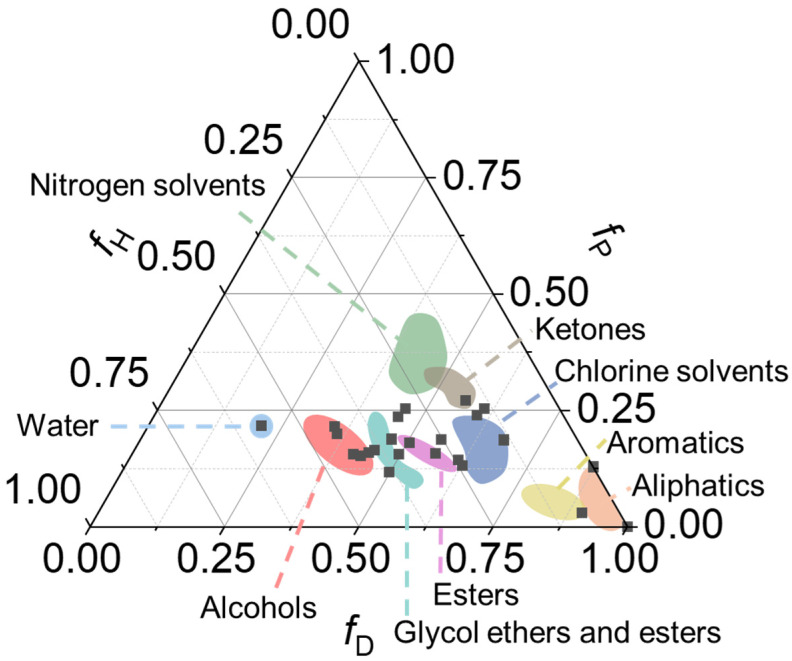
Triangular Teas diagram (plot of fractional solubility parameters) illustrating the match between classes of solvents used in the traditional restoration practice (colored areas) and some of the fully “green” solvents (black dots) recently reviewed by Casini et al. [7]. Adapted from [7]. Copyright © 2023, The Authors.

Indeed, the “green” metrics of the solvent employed for gel matrices preparation is a key factor, requiring detailed categorization that can be performed via the development of precise life cycle assessments [99]. Based on the combination of a list of “green” score from industrial and academic institutions, Casini et al. reported “global” scores for solvents and surfactants of interest in Cultural Heritage conservation and beyond [7]. Overall, solvents with excellent “green” metrics comprised several alcohols, esters, ethers, hydrocarbons, ketones, and terpenes, covering a wide range of properties such as dielectric constant, molar volume, and solubility parameters that are useful to assess the solubility of polymers/thickeners [39]. In particular, some of these “green” solvents match, or are close to, regions of the Teas diagram (plot of fractional solubility parameters) related to ketones, chlorine solvents, alcohols, esters, and aliphatic and aromatic compounds commonly employed in the traditional restoration practice (Figure 4).

In addition, recent promising classes of “green” solvents for potential confinement in sustainable organogels include fatty acid methyl esters (FAMEs) and deep eutectic solvents (DESs). FAMEs are derived from renewable sources and exhibit biodegradability and low toxicity, with lower-polarity FAMEs, also showing reduced evaporation rates that decrease risks to the environment and operators [100]. Recently, Biribicchi et al. showed that these solvents can remove beeswax and microcrystalline wax from stone and bronze artworks [100]. Longer-chain FAMEs are safer than their shorter-chain counterparts, which involve higher risks of swelling paint layers [101]. On the other hand, retention of longer-alkyl chain FAMEs in porous matrices, such as marble, is being investigated (e.g., by NMR relaxometry [102]) to understand possible long-term drawbacks for the artworks. Effective removal of wax coatings occurred using DESs, highlighting the potential of these “green” solvents for cleaning works of art [31].

The promising impact of these novel solvents calls for sustainable methodologies to mitigate their penetration and retention in porous artwork substrates. Overall, the broad solubility area covered by these fluids requires confining systems with tunable hydrophilicity/hydrophobicity and optimal uptake–release behavior. Additionally, the novel organogels should be formulated from renewable resources to comply with green metrics. In this regard, the life cycle assessment of the organogels’ constituents represents a first step for their development since it enables a first screening of suitable polymeric materials to be employed for organogel formulation [103,104]. Among the polymeric classes capable of conferring good green metrics, biopolymers are the most intriguing given their natural occurrence [105] and are already established for applications such as biomedics [106], packaging [107], and waste management [108].

Biopolymers with good hydrophilicity are already involved in the preparation of hydrogels agriculture, medicine, and even Cultural Heritage preservation [98,109,110]. Polysaccharide-based materials such as agar, chitosan, gellan, and xanthan gum are among the most studied biopolymers for the development of thickening agents [22,111], while chemical hydrogels can be obtained by polymerization and crosslinking of bioderived polymers and monomers [112,113,114]. In addition, the research of sustainable production of polymers could enable classic petroleum-based materials to be obtained in a more sustainable fashion. For instance, poly(vinyl alcohol), a long considered non-renewable polymer can be produced from sustainable resources [115], enabling the classification of well-known hydrogels for Cultural Heritage conservation as eco-friendly [53,116]. In contrast, the use of biopolymers for the formulation of organogels, complementary to hydrogels for highly water-sensitive surfaces [7,76,117], is still in its infancy.

In this regard, polyhydroxyalkanoates could represent a suitable choice for the preparation of green physical organogels since they can be directly produced from bacterial fermentation and can feature a wide variation of properties [118,119,120]. Until now, a series of polymer thickeners based on polybutyrate solutions has been developed for the cleaning of oil paintings [79,80] and bronze and iron artifacts [81,82], overall resulting in ideal candidate for the cleaning of water-sensitive surfaces [83,85]. In particular, physical organogels comprising poly-3-hydroxybutyrate (PHB), triethyl citrate, and γ-valerolactone (GVL) have shown potential to remove varnishes on oil paintings.

**Figure 5:**
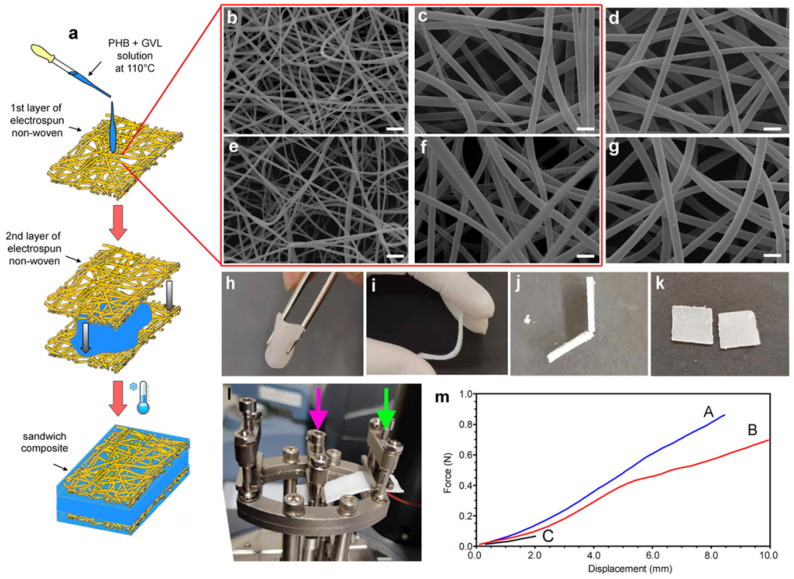
A scheme of the preparation (**a**) of PA6,6/PHB–GVL and PVA/PHB–GVL sandwich-like composites, showing also SEM pictures of PA6,6 (**b**,**c**) and PVA (**e**,**f**) fibers at different magnifications. SEM pictures of PA6,6 (**d**) and PVA (**g**) fibers after immersion in GVL for 1 min at 110 °C and dried at RT are also shown. The sandwich-like composite (**h**,**i**) and PHB–GVL gel (**j**,**k**) show different mechanical resistance to bending. Pictures (**l**,**m**) show the single cantilever configuration for sample mounting and force–displacement curves of PA6,6/PHB–GVL composite (blue), PVA/PHB–GVL composite (red), and PHB–GVL gel (black). Scale bar: 6 μm (**b**,**e**); 2 μm (**c**,**d**,**f**,**g**). Reproduced from [83]. Copyright © 2020 American Chemical Society.

The design of the new cleaning systems has been performed by varying the PHB/GVL ratio to tune viscosity and storage/loss moduli that are connected to the materials’ adaptability to complex surfaces. The first application resulted on the removal of the dammar layer from aged oil paints, without affecting the pictorial layer and leaving scarce amounts of the GVL solvent, which, however, having a high boiling point (205 °C), required complete removal to avoid risks connected to contamination. Tests on a 17th-century Italian paint resulted in the removal of terpene varnish layers. Interestingly, the authors also outlined the good recycling and biodegradability features of the new formulation, opening for their application for different conservative purposes. PHB-based formulations prepared with GVL or with benzyl alcohol showed higher cleaning capability than traditional solvent–gel formulations, with cleaner and more efficient performance [80]. The combination of PHB-GVL gels with PVA or polyamide-6,6 fibers was also tested for the removal of varnish from paintings [83], including a PHB-GVL layer between two outer layers of hydrophilic polymers, and we also took advantage of the synergistic combination of softer polymer thickener and stiffer PVA or polyamide-6,6 to further improve the varnish removal (Figure 5).

**Figure 6:**
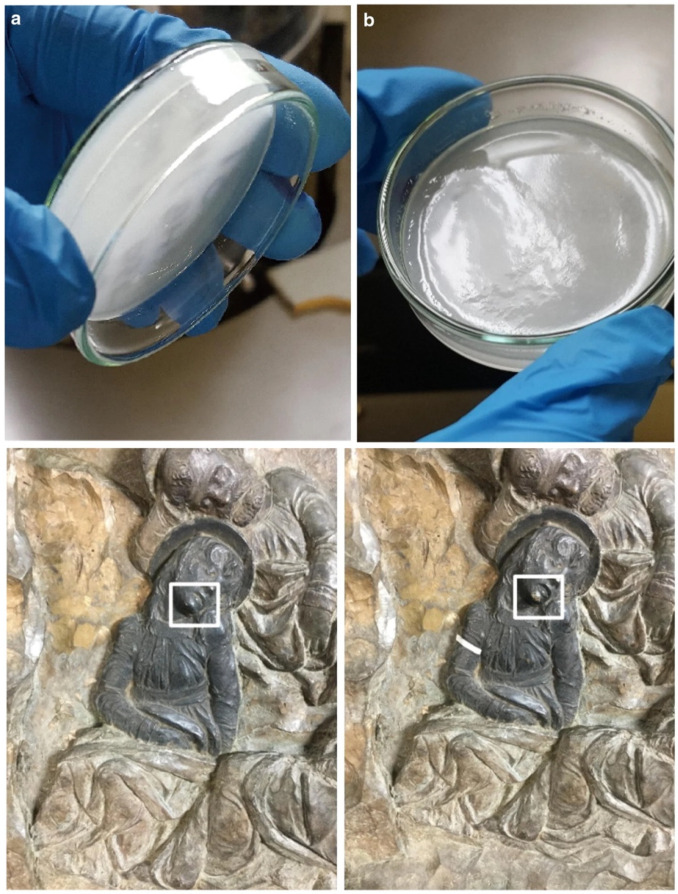
(**Top**). Visual aspect of PHB-BD/DMC (3:1) and PHB-BD/DMC (1:1) organogels (**a**,**b**). (**Bottom**). A detail of “Pulpito della Passione” (Donatello, 1460, Florence) before (**left**) and after (**right**) the cleaning using the PHB-DMC/BD (3:1) system. Reproduced from [81]. Copyright © 2019, The Authors.

Representing an aliphatic polyester with moderate hydrophobicity, PHB can, in principle, form physical gelled systems with a vast list of organic, sustainable solvents. For instance, the replacement of GVL with biodiesel and dimethyl carbonate can tune the hydrophobic/hydrophilic character of the gels for tailored applications. In fact, biodiesel is formed by a mixture of methyl esters of fatty acids [121], resulting in high lipophilicity, but blends with dimethyl carbonate can soften PHB. Tests with these systems allowed the removal of aged beeswax coatings from a 15th-century Donatello’s artwork [81] (Figure 6). PHB formulations were furthermore implemented for the single-step removal of varnish layer and corrosion products from metal surfaces. To this aim, a PHB system with porous interconnected porosity was formulated with ethyl lactate and deferoxamine B for the combined removal of iron corrosion salts and Paraloid B72, resulting in optimal removal [82]. The naturally occurring deferoxamine B (DFO) and Ethylenediamine-N,N′-disuccinic acid (EDDS) are also possible additional components as ligands to clean iron, copper (brass), and silver [84]. This application can be of particular interest for metal artifacts where prompt removal of degradative processes avoids the development of autocatalytic processes, as in the bronze disease [122,123]. Overall, ATR-FTIR microscopy and GC-MS confirmed high cleaning performance on mock-ups 24 h after a 5 min treatment [81].

Among natural-polymer-based materials, a physical gel based on agar or PVA, choline chloride, and urea was recently prepared in water/ethanol deep eutectic solvent (1 to 1 mixture) [85,86]. Such a formulation is based on the formation of deep eutectic solvent-based gel [124], comprising the eutectic mixture water/ethanol in the three-dimensional structure of agar/PVA and choline chloride. The physical interactions between the deep eutectic solvent and PVA are hydrogen bonds, as observed via ^1^H NMR and ATR-FTIR. The gelled samples enabled a more controlled diffusion of the clearing agent on varnish layers with respect to the pure solvent, resulting in a good control over the removal of patinas related to dirt or to Paraloid B72 varnish. Applications included the removal of Paraloid B72 from tempera or of an aged acrylic coating from mural paintings, while physical organogels from choline chloride, urea, and agar effectively removed proteinaceous varnishes from hydrophobic and hydrophilic surfaces (mock-ups with egg or rabbit glue coatings). Micro-FTIR confirmed the good cleaning efficacy of these gels on hydrophilic tempera mock-ups (egg as binder) coated with Paraloid B72 [85]. ATR-IR and optical microscopy showed that the choline chloride and urea agar gel can efficiently remove proteinaceous varnishes on both hydrophobic and hydrophilic surfaces [116]. Traces of choline chloride and urea were detected but were removed by applying an EtOH–H_2_O/agar gel for ca. 10 s.

Cellulose-ether-based organogels are also possible networks for the uptake of methyl myristate and isopropyl palmitate, replacing or minimizing the use of ligroin [87]. Hansen solubility parameters (HSPs) together with hydrophilic–lipophilic balance (HLB) values can predict chemical affinity among gel components and solvents, aiding the design of stable formulations. The apolar phase was selected by calculating relative energy difference number (RED) using the HSPs of various cellulose ethers, leading to Klucel-G as the thickener. Surfactants can then ensure compatibility between the apolar phase (ligroin) and the polar phase for long-term stability, resulting in five options with different HLB values: cetyl alcohol, Tefose 63, PEG-100 stearate, a mix of Tween 20 and Span 80, and Span 60. While HLB is helpful for emulsion formulation, it is not sufficient on its own for selecting the optimal surfactant.

Based on the evaluation and the tuning of HLB and of Hansen solubility parameters, such materials enabled removal from metallic substrates (e.g., copper) of different varnish/patinas based on microcrystalline waxes, Paraloid B44 acrylic resin, and Incral protective varnish. Additionally, hydroxypropylcellulose-based gels in ethanol/ligroin mixture yielded effective cleaning of 19th-century canvas paintings, with optimum removal of aged varnishes (e.g., shellac) [88]. FTIR analysis evaluated the effectiveness in removing various protective coatings, the level of cleaning achieved, and the presence of residues and confirmed the effective removal of aged shellac varnish from the painting surface [88]. Shellac’s high hydrogen-bonding capability plays a significant role in the solvation mechanism [88]. Gels based on methyl myristate and isopropyl palmitate showed promise as “green” alternatives to ligroin, although, in some cases, they exhibited lower selectivity in removing the protective coatings.

In contrast with the physical PHB and cellulose-derivative organogels previously described, chemical organogels benefit from a higher mechanical stability but often have higher stiffness and lower surface adaptability. Chemical organogels from bioderived resources are thus an open field in conservation science.

A possible synthetic approach is photopolymerization. Isosorbide, pyrogallol, and limonene-containing gels were produced accordingly via thiol-ene polymerization. The gels’ synthetic processes and structural features were evaluated, highlighting moderate swelling capability in dimethyl carbonate (DMC, up to 54% *w*/*w*), ethyl acetate (EA, up to 30% *w*/*w*), acetone (up to 18% *w*/*w*), and limonene (up to 14% *w*/*w*). The gels exhibited a greater tendency to absorb relatively polar solvents (EA, DMC, and acetone) than non-polar ones (limonene and hexane). Because these gels are more highly crosslinked than similar systems reported in the literature, they also showed lower swelling percentages. The gelled systems obtained with dimethyl carbonate were employed for the removal of dammar/linseed oil varnishes from an easel painting [89]. All peaks related to the varnish disappeared from the FTIR spectra, demonstrating high efficiency for dammar/linseed oil removal. However, it must be noted that these gels might be too aggressive and could remove part of the original binder. Future studies will test the gels on aged samples with known binders.

Another intriguing possibility to obtain chemical organogels is the use of triglyceride mixtures such as castor oil (CO). CO represents a renewable, nonedible natural product finding application in medicine, cosmetics, and lubricant industries [125]. As a result, the use of CO can be particularly attractive for the development of new sustainable gels and rubbers of interest in and beyond Cultural Heritage preservation [76]. The preparation of gels and rubbers from CO is often obtained by means of urethane chemistry [126]. Over the last years, such an approach has allowed the preparation of new organogels for preventive and remedial conservation. For instance, CO polymerized with polyisocyanates, and including inorganic fillers such as zinc oxide, yields a series of rubbers capable of binding VOC pollutants (e.g., acetic acid) produced by frames or even art materials, which otherwise promote autocatalytic degradation of artifacts [127,128]. Representing polyurethane-based materials obtained with cleavable ester bonds (from the triglyceride structure), such rubbers have good biodegradability and compostability, resulting in degradation of the materials [90], e.g., weight loss in soil of 25% after 150 days. Water absorption enlarges the surface area available for microbial attack and can promote hydrolysis of ester groups within the PU network, thereby improving the degradation rate.

Organogels based on castor oil polyurethanes (CO-PUs) are also new promising materials for the removal of varnishes from works of art [91]. The new polyurethanes were obtained through a fully solvent-free synthesis, with various amounts of crosslinking agent, with or without the use of polyethylene oxide additives, and resulted in dry systems (rubbers) according to sustainable synthetic protocols. To rationalize the differences in swelling, Hansen solubility parameters (HSPs) can predict to some extent polymer–liquid interactions. HSPs for gel components and for pure solvents were taken from HSPiP software (Hansen solubility parameters in practice), while gel HSPs were calculated from those of pure components by combining them according to their volume fraction in the uncured mixtures, as is commonly done for solvent blends.

**Figure 7:**
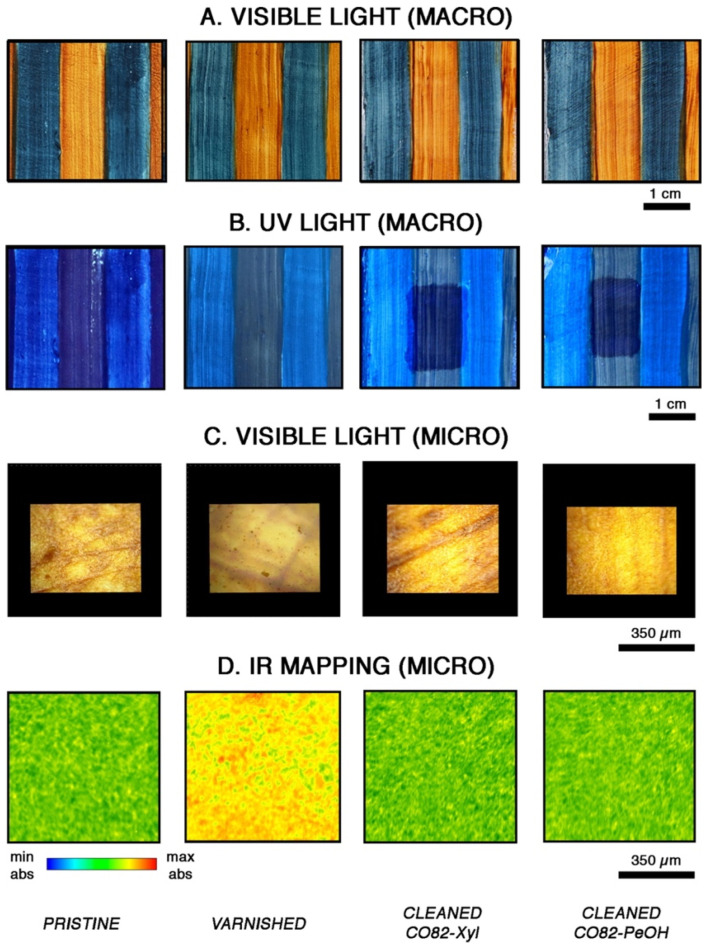
Pictures of watercolor canvas painting mock-ups acquired under visible light (**A**) and UV light (**B**) on the untreated sample (pristine) after the application and aging of the acrylic–ketonic varnish (varnished) and following treatment with castor-oil-based organogels swollen in xylene and pentanol. Optical microscope pictures of the mock-ups (**C**). Two-dimensional FTIR imaging of the same samples obtained by mapping the 1860–1685 cm^−1^ region, where the characteristic absorption of the acrylic–ketonic varnish falls (**D**). Reproduced from [91]. © 2023 The Authors. Published by Elsevier Inc.

In particular, rheology and neutron scattering elucidate the swelling of the dry systems with p-xylene and pentanol, providing structural information prior and after swelling. The analysis revealed that the gelled systems confine the solvents in their polymeric mesh, forming a highly retentive system with controlled release of the fluids. Selected gel samples removed an acrylic–ketonic varnish from watercolor canvas paints and resulted in complete and time-effective varnish removal as demonstrated by FTIR 2D imaging (Figure 7). A great advantage of this class of organogels is their sustainable and easily scalable synthetic process [90]: The amount of waste in the process is close to 0, similar to oil refining processes (E-Factor < 0.1), and thus much better than the bulk chemical sector of typical polyurethane production (E-Factors ranging from 1 to 5). The mass intensity (MI) is close to the ideal limit value (1), and the reaction mass efficiency (RME%) is ca. 98% (where 100% is the ideal value), owing to minimal loss of reagents during mixing and pouring.

**Figure 8:**
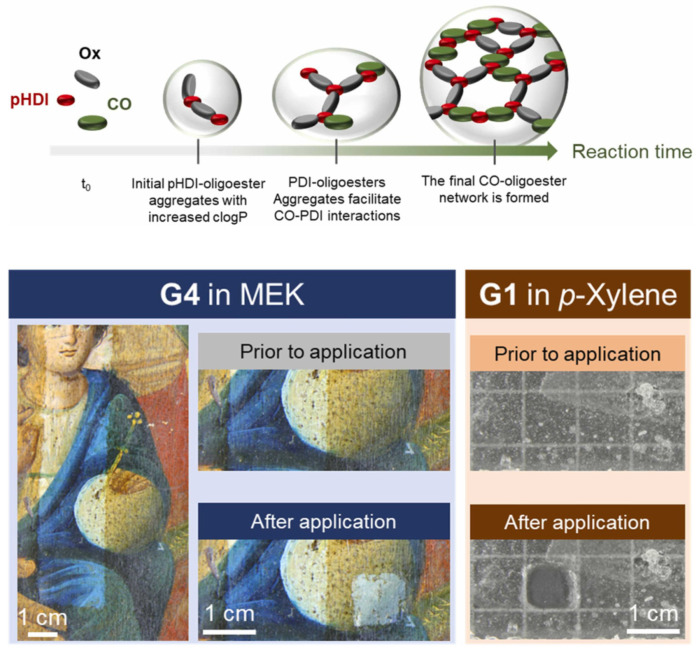
(**Top**). A scheme of the reaction between castor oil, oligoesters (Ox), and isocyanates (pHDI) to yield castor oil–oligoester polyurethane gel network, proposed on the basis of rheological and NMR data. (**Bottom**). Cleaning tests using castor oil–oligoester gels to remove a polyhydroxyalkanoate resin from a painted wood panel (**left**) and beeswax from a glass slide (**right**). Reproduced from [92]. © 2024 The Authors. Published by Elsevier B.V.

Aiming to vary the structural features of the CO-PU gels, a series of oligoester additives were prepared and employed for the preparation of new gelled materials [76]. The new oligoesters were designed to have similar molar mass but controlled variation of chemical composition and hydrophobicity. Starting from the study of the features of each oligoester, structure–property evaluation of the oligoesters’ thermal, mechanical, and hydrophobic features connected to the final CO-PUs’ properties via a new method for the evaluation of octanol/water partition coefficient (*logP*). Interestingly, the evaluation of *logP*, which gives indirect indications on hydrophobicity, gave excellent results for understanding and predicting the COPUs’ properties during synthesis and as gelled materials. The type of oligoester additive employed in the gels feasibly regulates their rheological and thermal properties, degree of crystallinity, swelling compatibility, maximum swelling capability, and swelling process [76,92]. Two organogels of different hydrophobicity swelled in p-xylene and acetone, respectively, and then removed polymer coatings and wax from frosted glass slides simulating hydrophilic substrates. First, it was first confirmed that the Teas solubility parameters of the two solvents fall within the swelling regions of the target coatings. Then, the final oligoester-containing solvent-swollen COPU organogels successfully removed coatings such as wax, Paraloid B72, and polyhydroxyalkanoate resins (Figure 8).

In the castor oil–oligoester polyurethane gels, the maximum swelling percentage varied with solvent polarity and the hydrophobicity of the oligoester additive. For moderately polar solvents such as acetone and MEK, gels containing the less lipophilic additives reached the highest uptake (relative weight increase ≈ 2.24 for acetone, ≈3.17 for MEK), while the most hydrophobic gel showed lower values (relative weight increase ≈ 1.97 and ≈2.82, respectively). In contrast, for the more hydrophobic p-xylene, the addition of the hydrophobic additive achieved the highest swelling (≈2.80), while hydrophilic additives achieved the lowest (≈2.36). Ethanol, acetone, MEK, and DEC exhibited a predominantly Fickian diffusion mechanism in the gels, with Fickian contribution highest in more hydrophobic gels and decreasing as the oligoester *clogP* decreased; p-xylene showed the opposite trend. This behavior reflects the interplay between gel network hydrophobicity and solvent *logP*: when solvent *logP* is lower than the oligoester’s, diffusion dominates, whereas with more hydrophobic solvents like p-xylene, chain relaxation becomes more significant.

In addition to CO-PU gels, a new class of poly(urethanes-co-oxazolidones) organogels based on the environmentally friendly CO epoxide has been recently developed and characterized [93]. These new materials benefit from the reactivity of epoxides with isocyanates, resulting in the formation of oxazolidone bonds. The addition of such moieties was traced by RAMAN spectroscopy and resulted in the formation of denser dry polymeric networks with increased stiffness. These gels, swollen for 30 min in “green” solvents, exhibited a greater decrease in G’ with MEK or acetone than with diethyl carbonate, indicating their suitability for controlled swelling and adhesive/sealant removal. Swelling depends on the ratio between CO and epoxidized CO (EpCO) used in the synthesis: EpCO-rich gels, with higher crosslinking density from oxazolidinone linkages, restrict solvent uptake, while unreacted epoxide rings enhance solvent–polymer affinity; in CO-only gels, lower crosslinking facilitates solvent entrapment, and increasing EpCO shifts the balance toward reduced swelling. The new systems can then swell in selected organic solvents such as diethyl carbonate and 2-butanone, resulting in an increase in their elastic module. These features make the new organogels potential materials for the development of sustainable sponges, rubbers, adhesives, and sealants in different applications, included artworks’ cleaning.

Table 1 summarizes the mechanical properties, loadable solvents, cleaning efficiency, and tested case studies for the new bio-derived organogels discussed in this session, including systems like rice bran oil and cinnamic acid, some vegetable/mineral oil organogels, or the COPUOxazolidones, which have potential use in Cultural Heritage. Overall, organogels from renewable resources with high “green” metrics represent a new, fundamental class of materials for Cultural Heritage preservation, which in principle can surpass traditional petrol-based materials. Given the possibility to incapsulate and release “green” solvents, such materials could represent a sustainable alternative to well-established cleaning systems, complementary to hydrogels in the palette of Cultural Heritage conservation tools.

## 4. Conclusions and Future Perspectives

Organogels, i.e., gelled networks capable of confining organic solvents with average or low polarity, constitute an example of materials for Cultural Heritage preservation, where soft matter and colloids can devise solutions useful to multiple fields. Research in organogels for the cleaning of works of art is still widely unexplored as the focus is shifting from petroleum-based and synthetic polymers to bio-derived sustainable materials. This effort goes in parallel with renewed interest in “green” solvents such as alkyl carbonates, fatty acid methyl esters, and deep eutectic solvents, which have intriguing physico-chemical properties but need confinement in retentive matrices for many practical applications. Starting from physical networks based on polyallylamine, chemical polymeric gels were then devised using methacrylate monomers, complemented by systems as viscous polymer dispersions of polyvinyl acetate crosslinked with borax, or polydimethylsiloxane organogel sponges. However, while these tools improved greatly on the traditional solvent blends and thickeners used in the restoration practice, compliance with the new recommendations on sustainable materials is progressively pushing the development of bio-inspired and bio-based gelled systems. In this context, physical gels were recently realized with poly-3-hydroxybutyrate (PHB), while chemical networks were synthesized using castor oil (CO) and its derivatives. These two applications are representative of the potential that dedicated research in Cultural Heritage holds to develop novel bio-based materials of wide interest since both PHB and CO are central components in bioplastics or degradable biomaterials [126,129]. Polymeric low-molecular-weight gelators capable of entrapping low polar solvents represent another class of organogels of high interest [130]. Examples comprise cholesterol, fatty acids [131], amino acid, and saccharide-based systems [132]. Such systems have not yet been employed for Cultural Heritage restoration and represent promising novel candidates to inspire future research in the field.

Finally, an important perspective regards the incorporation in organogels of bio- and green surfactants to tune their porosity, mechanical properties, and overall performance in the cleaning of artworks. Current recommendations in the surfactant industry are to progressively replace petroleum-based surfactants or compounds that show environmental issues (e.g., alcohol ethoxylates) using instead bio-derived non-ionic surfactants [133].

It must be remarked that quantitative life cycle assessment (LCA) and environmental impact analysis are still largely unexplored for these new, promising classes of organogels in Cultural Heritage conservation. In addition, carbon footprint comparisons between bio-based organogels and conventional materials are essential and should be the target of investigations in the near future. Quantitative data on biodegradability and associated time cycles should be provided to allow a clearer evaluation of the environmental impact of these new materials. For instance, some available information on the new class of castor oil organogel polyurethanes [125] point at very good “green” metrics and scalability of their synthetic process, e.g., low energy/cost, no solvent needed, very low E-factor, and almost ideal mass intensity (MI) and reaction mass efficiency (RME%). Similar sustainability evaluations should be performed for all the newly proposed bio-derived systems in Cultural Heritage conservation.

Overall, the combination of a high safety and environmental profile with enhanced and controlled removal of unwanted layers proves novel organogels’ potential and, in some cases, already actual examples of Safe and Sustainable by Design practice, which set a new standard in materials design. An important perspective and additional challenge regard the presence of the regulatory barriers and AI-based formulation tools, which are still largely unexplored in the synthesis and application of bio-derived organogels for Cultural Heritage preservation. Regulatory barriers certainly include the necessity of using feedstock with certified provenance as bio-based building blocks for the synthetic processes to make sure that this aspect is well included in the LCA of the new gel materials. AI and machine learning certainly offer large potential for integration in the formulation processes. Overall, research in organogels for cleaning artifacts is a valid example of increasing links between soft matter in Cultural Heritage preservation and technological sectors such as the cosmetic, food, healthcare, and pharmaceutical industries, fostering sustainable socioeconomic regrowth.

## Figures and Tables

**Table 1:** Summary of traditional polymer or highly viscous polymer dispersions (HVPDs) and organogels proposed for Cultural Heritage conservation or organogels with potential in the field.

Code	Type	Mechanical Properties (Rheology, *G’*)	Solvents	Cleaning Efficiency	Case Studies	Ref.
**Solvent gel**	Thickened polymer dispersion	100–120 Pa	Alcohols, ketones, esters, aliphatic and aromatic hydrocarbons	FTIR, SEM, chromatography techniques	Widely spread in traditional practice	[7,46,47,48,49,50]
**Traditional organogel formulations**						
PMMA	Chemical gel	Hard gels	MEK, ethyl acetate, cyclohexanone, butyl acetate, DEC	FTIR	Paper artworks, canvas paintings	[54,58,59,60]
PAA-CO2	HVPD	>100 kPa	Acetic acid with methanol, ethanol-octanol	FTIR	Canvas painting, wood	[56,57]
PVA-borax	HVPD	Max ca. 1 kPa	Dimethyl sulfoxide, 2-ethoxyethanol, dimethylformamide, methanol, 1-methyl-2-pyrrolidinone, tetrahydrafuran, 95:5 ethanol/water	NMR, FTIR	Gilded wood, canvas paintings, marble	[61,62,64,65]
PDMS organogels	Chemical gel	>100 kPa	Acetone, DEC, DMSO, ethyl acetate, ethanol, water, isopropanol, cyclohexane	FTIR	Frescos, canvas paintings	[68]
**Bio-derived organogel formulations**						
COPU-Ox	Chemical gel	>80 kPa	Acetone, MEK, p-xylene	N/A	Glass	[76]
Rice bran oil and cinnamic acid	Physical gel	1–100 kPa	N/A	N/A	N/A	[77]
Vegetable and mineral oil	Physical gels	Vegetable max 10 kPa, mineral max 3000–6000 Pa	N/A	N/A	N/A	[78]
PHB	Physical gels	20–200 kPa, or 1–300 kPa	G-valerolactone, biodiesel/dimethyl carbonate, ethyl lactate	ATR, GC-MS	Bronze, canvas paintings	[79,80,81,82,83,84]
PVA, ethylene glycol and choline chloride	Physical gels	80–200 kPa	Ethylene glycol	ATR	Tempera, mural paintings	[85]
Choline chloride, urea and agar	Physical gels	Ca. 300 kPa	1:1 water ethanol mixture	ATR	Oil and tempera	[86]
Cellulose ethers	Physical gels	N/A	Methyl myristate, isopropyl palmitate, Ligroin, ethanol	FTIR	Copper, canvas paintings	[87,88]
Isosorbide, pyrogallol, and limonene	Chemical gels	N/A	Acetone, ethyl acetate, DMC, hexane, limonene	FTIR	Canvas paintings	[89]
COPUs	Chemical gels	>20 kPa	p-xylene, pentanol	FTIR 2D Imaging	Canvas paintings	[90,91]
COPU-Ox	Chemical gel	>80 kPa	Acetone, MEK, p-xylene	N/A	Canvas paintings	[92]
COPUOxazolidones	Chemical gels	>20 kPa	DEC, MEK, Acetone	N/A	N/A	[93]

## Data Availability

No new data were created or analyzed in this study.
